# Prevalence and predictors of analgesic use during early pregnancy in a Brazilian population

**DOI:** 10.3389/fphar.2026.1730483

**Published:** 2026-03-05

**Authors:** Marcella Tapias Passoni, Mariana Regina Rompkovski, Vitória Aline Santana Rios, Daniele Cristine Krebs Ribeiro, Amanda Atuati Maltoni, Carla Giovana Basso, Sara Emilia Lima Tolouei, Juliana Machado Franco, Bianca Manfroi da Silva, Anderson Joel Martino-Andrade, CARES Study Group

**Affiliations:** 1 Laboratory of Reproductive Toxicology, Department of Pharmacology, Federal University of Paraná, Curitiba, Brazil; 2 Laboratory of Animal Reproductive and Endocrine Physiology, Department of Physiology, Federal University of Paraná, Curitiba, Brazil

**Keywords:** analgesics, dipyrone, ibuprofen, paracetamol, pharmaceuticals, pregnancy

## Abstract

**Introduction:**

Although the use of analgesics is generally not recommended during pregnancy, several studies have reported a high prevalence of use among pregnant women. In this study, we assessed the prevalence of early pregnancy use of analgesics in a Brazilian population, as well as potential sociodemographic and lifestyle predictors.

**Methods:**

Pregnant women up to 16 weeks of gestation (N = 275) were recruited in Curitiba, Brazil, and specifically asked about the use of paracetamol, dipyrone, ibuprofen, acetylsalicylic acid, and diclofenac, including common brand names and indications.

**Results:**

The consumption of any analgesic up to the point of recruitment was reported by 61.5% of women, most commonly for the treatment of headaches. Paracetamol was the most used analgesic (55.3%), followed by dipyrone (13.5%) and ibuprofen (12%), and the use of more than one analgesic was reported by 18.5% of participants. The self-reported health status was a significant predictor. Women reporting fair/poor health were more likely to use any analgesic and paracetamol than those who reported good/excellent health status (OR = 3.05; 95% CI = 1.44–6.50). Among paracetamol users, women reporting the consumption of paracetamol and other analgesics ingested more paracetamol pills than those participants who reported the use of paracetamol-only. Similarly, the use of pharmaceuticals other than analgesics was also positively associated with the heavy use of paracetamol (OR = 3.70; 95% CI = 1.08–12.74).

**Discussion:**

Overall, the high prevalence of analgesic use during early pregnancy, particularly paracetamol and the combination of different analgesics, highlights the need for further research across different global regions and their potential implications for maternal and fetal health.

## 1 Introduction

Mild analgesics and non-steroidal anti-inflammatory drugs (NSAIDs) are among the most frequently used pharmaceuticals worldwide, usually as self-medication (Beckhauser et al., 2010; Dale et al., 2015; Sarganas et al., 2015; Arrais et al., 2016; da Silva Dal Pizzol et al., 2019; Moreira de Barros et al., 2019). Mild analgesics, represented by drugs such as paracetamol and dipyrone, are compounds used to relieve mild to moderate pain and often reduce fever, but do not possess significant anti-inflammatory activity. In contrast, non-steroidal anti-inflammatory drugs (NSAIDs), exemplified by acetylsalicylic acid, ibuprofen, and diclofenac, provide pain relief, fever reduction, and anti-inflammatory action (Grosser et al., 2018). In this study, mild analgesics and NSAIDs will be collectively referred to as analgesics unless otherwise specified.

The widespread availability of analgesics as over-the-counter drugs and relatively low cost contribute to extensive use in the general population, including vulnerable groups such as pregnant women. During pregnancy, analgesics are commonly used to manage pain, fever, and inflammatory conditions, which are frequently reported throughout gestation (Ostensen and Skomsvoll, 2004; Black et al., 2019). Epidemiological studies from different countries consistently report a high prevalence of analgesic use among pregnant women, including occasional concomitant use of more than one compound (McKenna and McIntyre, 2006; Rebordosa et al., 2008; Kristensen et al., 2011; Philippat et al., 2011; Thorpe et al., 2013).

Analgesics and NSAIDs are known to cross the placental barrier (Wunsch et al., 2003), and their gestational use is generally contraindicated by regulatory bodies unless the benefits outweigh the risks to the mother and fetus. Paracetamol is generally considered safe when used with medical supervision, while regulatory agencies, such as the FDA - Food and Drug Administration. 2020, the EMA - European Medicines Agency, 2022 and the Brazilian Health Regulatory Agency (ANVISA, 2010) advise avoiding maternal exposure to NSAIDs, especially from mid-pregnancy onwards. Prenatal exposure to these drugs, especially after 20-weeks gestation, has been associated with adverse fetal outcomes, including spontaneous abortion, premature closure of the ductus arteriosus, impaired fetal renal function, oligohydramnios, and delayed labor (Schoenfeld et al., 1992; Rathmell et al., 1997; Antonucci et al., 2012; Servey and Chang, 2014; Li et al., 2018; Ying et al., 2021; Dathe et al., 2022; Tain et al., 2025). Paracetamol is the most frequently used analgesic during pregnancy, largely because it is not associated with many of these adverse effects. Estimates indicate that approximately 50%–70% of women use paracetamol at some point during gestation (Werler et al., 2005; Rebordosa et al., 2008; Kristensen et al., 2011; Thorpe et al., 2013; Lind et al., 2017; Bertoldi et al., 2020; Navarro-Lafuente et al., 2021). Although paracetamol is generally considered appropriate when used as medically indicated, accumulating evidence suggests potential associations between prenatal exposure and outcomes such as neurodevelopmental, respiratory, urogenital, and reproductive disorders. (Jensen et al., 2010; Kristensen et al., 2011; Snijder et al., 2012; Liew et al., 2014; Aminoshariae and Khan, 2015; Sordillo et al., 2015; Masarwa et al., 2018; Bauer et al., 2021; Nielsen et al., 2025). However, the available data remain inconclusive, and some studies have found no evidence of associations between prenatal paracetamol use and these outcomes (Ahlqvist et al., 2024; Sheikh et al., 2025).

In addition to paracetamol, other analgesics are also widely used by pregnant women, but the pattern of use vary substantially across regions. In several countries, including Brazil, dipyrone (metamizole) is frequently used during pregnancy, often ranking second after paracetamol, despite the overall recommendation to avoid this drug during gestation due to increased risks of adverse fetal outcomes, smilar to those reported for NSAIDs (Mengue et al., 2001; Fonseca et al., 2002; Da Silva Dal Pizzol et al., 2009; Dathe et al., 2017; Dathe et al., 2022). In contrast, in countries where dipyrone is banned or subject to strict regulatory restrictions, such as the United States and much of Western Europe, ibuprofen is more commonly reported as the second most frequently used analgesic during pregnancy (Kristensen et al., 2011; Thorpe et al., 2013). These regional differences highlight the importance of country-specific data when assessing prenatal exposure to analgesics.

Beyond the documented adverse outcomes of prenatal exposure to analgesics and NSAIDs (Antonucci et al., 2012; Ying et al., 2021; Tain et al., 2025), many other potential adverse consequences have been under scrutiny. Recent epidemiological and experimental studies suggests that several analgesics and NSAIDs, including paracetamol, ibuprofen, acetylsalicylic acid, indomethacin, diclofenac, and dipyrone may interfere with endocrine signaling pathways. These suspected endocrine disrupting properties have been associated with reproductive effects in humans and experimental models of both sexes across different life stages (Kristensen et al., 2011; 2012; Albert et al., 2013; Mazaud-Guittot et al., 2013; Dean et al., 2013; Holm et al., 2015; 2016; Ben Maamar et al., 2017; Lind et al., 2017; Leverrier-Penna et al., 2018; Passoni et al., 2018; Krebs Ribeiro et al., 2020; Passoni et al., 2022). In addition, some studies indicate that prenatal exposure to certain analgesics may associated with intergenerational effects on fertility (Dean et al., 2016; Rossitto et al., 2019a; 2019b).

Despite the widespread use of analgesics during pregnancy and evidence of potential adverse health outcomes, data on early pregnancy exposure and its determinants remain limited in several regions, including Brazil. Updated population-based assessments are therefore needed to characterize current patterns of use, identify groups at higher likelihood of exposure, and direct and prioritize research efforts. In this context, the aim of the present study was to investigate the prevalence of analgesic use during early pregnancy in a Brazilian population and to identify sociodemographic and health-related predictors of exposure.

## 2 Methods

### 2.1 Study design and population

This study is part of an ongoing pregnancy cohort in the city of Curitiba (Brazil), the Curitiba Reproductive and Environment Study (CARES), designed to investigate exposure of pregnant women to environmental chemicals and pharmaceuticals, in particular analgesics and NSAIDs, and possible associations between such exposures and sociodemographic and lifestyle factors, as well as maternal-infant health outcomes. Here, we present data on the prevalence and predictors of analgesics and NSAIDs use in early pregnancy (≤16 weeks).

Pregnant women (N = 275) were recruited between March 2018 and March 2020 at ten public healthcare centers in Curitiba, representing the ten health districts of the city. At the beginning of the study, one of the health districts was dropped due to lack of accessibility. Participants who fulfilled the following inclusion criteria were enrolled in the study: pregnant women up to 16 weeks gestation who were registered in the Curitiba Maternal-Infant Healthcare Program, residents of Curitiba city, aged between 18 and 40 years, and whose pregnancy was classified as low-risk according to the Curitiba Maternal-Infant Healthcare Program guidelines.

Questionnaires were applied in the form of a face-to-face interview by a trained researcher linked to the study. The questions were structured to obtain information related to sociodemographic and lifestyle characteristics, including age, gestational age at recruitment, pre-pregnancy body mass index (BMI), prior pregnancies, self-reported health status, marital status, race, education level, income, smoking and alcohol consumption.

Participants were only interviewed after providing signed informed consent. CARES study protocols were approved by the Research Ethics Committees of the Health Sciences Sector of Federal University of Paraná (34820214.9.0000.0102 – n° 786.120) and the Municipal Health Department of Curitiba (34820214.9.3001.0101 – n° 926.727).

### 2.2 Data on analgesic use

The interview questionnaire at recruitment presented questions in sequence about indications and use of specific analgesics and NSAIDs. Firstly, women were asked whether they have experienced specific common symptoms since pregnancy begin such as headache, pain in the legs, back, stomach, or throat, flu, cold, fever, cramps, nausea, or others. Subsequently, women were specifically asked about the use of five main analgesics and NSAIDs to treat the previously mentioned symptoms or any other conditions: paracetamol, dipyrone, ibuprofen, acetylsalicylic acid, and diclofenac, one medication at a time and mentioning the name of the active ingredient and the most used brand names of these drugs in Brazil.

For each medication, women could answer yes or no as to whether they used such a drug. Those who responded positively were asked about the reason that led them to take the medication, being asked again about the specific symptoms or conditions mentioned above. Also, we asked about the number of pills they had taken since the start of pregnancy, regardless the dose amount in each pill. The use of drops was converted into the equivalent dose amount of one typical pill of paracetamol (500 mg), dipyrone (500 mg), or ibuprofen (400 mg). In addition, women were also asked if they used other pharmaceuticals, either of continuous or occasional use.

### 2.3 Statistical analyses

Descriptive statistics were obtained for the sociodemographic characteristics and pattern of analgesic use in the study population. Age, gestational age at recruitment, and pre-pregnancy BMI were analyzed as continuous variables, while all other variables were treated as categorical.

We used bivariate logistic regression analyses to examine the associations between analgesic use in early pregnancy and the sociodemographic and lifestyle factors assessed in this study. Dependent variables were the use of any analgesic (no/yes) and the use of paracetamol (no/yes). The variables listed in Table 1 were considered as potential predictors for analgesic use, in addition to recruitment site (Supplementary Table S1), and the use of pharmaceuticals other than analgesics (no/yes). We first conducted bivariate analyses to examine unadjusted (crude) associations between all these potential predictors and the use of any analgesics or paracetamol.

**TABLE 1:** Sociodemographic characteristics of study participants.

Variable	Mean ± SD or N (%)^a^
Number of participants	275
Age (years)	27.5 ± 6.1
Gestational age at recruitment (weeks)	11.8 ± 2.9
Pre-pregnancy BMI (kg/m^2^) (N = 268)	25.2 ± 5.0
Prior pregnancies	
* No*	152 (55.3%)
* Yes*	123 (44.7%)
Self-reported health status	
* Good/excellent*	222 (80.7%)
* Fair/Poor*	53 (19.3%)
Marital status	
* Married/living as married*	168 (61.1%)
* Living without a partner*	106 (38.5%)
*Missing*	1 (0.4%)
Race	
* White*	175 (63.3%)
* Other*	95 (34.5%)
* Missing*	5 (1.8%)
Education	
* Basic*	65 (23.6%)
* High School*	150 (54.5%)
* College or higher*	60 (21.8%)
Income	
* Up to 3 minimum wages*	165 (60.0%)
* 3–6 minimum wages*	79 (28.7%)
* > 6 minimum wages*	22 (8.0%)
* Missing*	9 (3.3%)
Smoking^b^	
* No*	221 (80.4%)
* Yes*	53 (19.3%)
* Missing*	1 (0.4%)
Drinking^b^	
* No*	182 (66.2%)
* Yes*	92 (33.5%)
* Missing*	1 (0.4%)

^a^ Data on continuous variables are presented as mean ± standard deviation and categorical data as counts and percentage.

^b^ yes/no since beginning of pregnancy.

Among paracetamol users, bivariate logistic regression analyses were also conducted to explore potential predictors of the categorized number of consumed pills since the beginning of pregnancy (1–19 pills/20 or more pills). The same set of independent variables described above was used, with the addition of the use of more than one analgesic (“paracetamol-only” vs. “paracetamol and other analgesics”) as a potential predictor. In this specific analysis, categorial variables with more than two categories were recoded into binary categories to account for the smaller sample size.

In the multivariable logistic regression models, all independent variables that resulted in p-values less than 0.25 in the bivariate (unadjusted) analyses were included in the final models. Multicollinearity among independent variables included in the final models was assessed using variance inflation factors (VIF) obtained from linear regression. The Hosmer-Lemeshow test was used to assess the goodness of fit, and models with p-values >0.05 were considered adequate. Associations between paracetamol use categories (“paracetamol-only” vs. “paracetamol and other analgesics”) and the number of consumed pills were additionally assessed using the Mann-Whitney U on ranked values. Missing data were not imputed, as the proportion of missing values was low. Participants with missing values in any variable included in each model were excluded from that specific analysis. All statistical analyses were conducted in IBM SPSS statistics 28.0.0.0 (Armonk, United States) and GraphPad Prism 9.1.2 (La Jolla, United States).

## 3 Results

### 3.1 Sociodemographic and lifestyle characteristics of study population

General data on our study population are shown in Table 1. A total of 275 pregnant women were recruited in nine municipal public healthcare units of Curitiba, Brazil (Supplementary Table S1). Mean age (± standard deviation) at enrolment was 27.5 ± 6.1 years and the mean gestational age was 11.8 ± 2.94 weeks, ranging from 3 to 16 weeks (median = 12.0 weeks). Most women were white (63.3%), married or living as married (61.1%), with low income (60.0%), basic or secondary education (78.1%), and were pregnant for the first time (55.3%). Smoking was reported by 19.3% of participants and 33.5% reported drinking at least one drink since beginning of pregnancy (Supplementary Table S2). Self-reported health status was good or excellent for 80.7% of enrolled women. The mean pre-pregnancy body mass index (BMI; ±standard deviation) was 25.2 kg/m2 (±5.0) and 41.5% of women were overweight or obese (BMI ≥25.0 kg/m2).

### 3.2 Analgesic use

Use of at least one analgesic from beginning of pregnancy up to study enrolment was reported by 61.5% of participants (Table 2). In this study we specifically asked about the use of paracetamol, dipyrone, ibuprofen, acetylsalicylic acid (AAS), and diclofenac. Paracetamol was by far the most used analgesic (55.3%), followed by dipyrone (13.5%) and ibuprofen (12%). AAS and diclofenac were used by only 2.5% and 1.5% of study participants, respectively.

**TABLE 2:** Use of mild analgesics and non-steroidal anti-inflammatory drugs in early pregnancy.

Variable	N (%)
Participants	275
Any analgesic	​
* No*	106 (38.5%)
* Yes*	169 (61.5%)
* Paracetamol*	152 (55.3%)
* Dipyrone*	37 (13.5%)
* Ibuprofen*	33 (12.0%)
* Acetylsalicylic acid (AAS)*	7 (2.5%)
* Diclofenac*	4 (1.5%)
More than one analgesic	51 (18.5%)
* Paracetamol and Dipyrone*	20 (7.3%)
* Paracetamol and Ibuprofen*	15 (5.5%)
* Paracetamol, Dipyrone, and Ibuprofen*	9 (3.3%)
* Other combinations*	7 (2.5%)

Most women reported sporadic use of individual analgesics (1-5 pills), but some participants indicated the use of higher amounts (Table 3). For paracetamol, we categorized the total amount of used pills in four categories and found that 11.8% of paracetamol users consumed 20 or more pills from beginning of pregnancy up to recruitment (Table 3). Among dipyrone users, five participants reported intravenous use. The most common reasons for taking analgesics were headache or headache combined to other reasons (Table 3). Use of more than one analgesic was reported by 51 women (18.5%). The use of paracetamol and dipyrone was reported by 20 women, which corresponds to 7.3% of study participants and 39% among those reporting the use of more than one analgesic, while paracetamol and ibuprofen was used by 15 women (5.5% of total participants and 29% of women that used more than one analgesic). We also asked women about use of pharmaceuticals other than analgesics and found that 48.8% of study participants used at least one drug, with the most common classes being antiemetics and antibiotics (Supplementary Table S3).

**TABLE 3:** Dose and reasons of paracetamol, dipyrone, and ibuprofen use in early pregnancy.

Variable	N (%)
Paracetamol (number of users)	152
* Total number of pills up to study enrollment*	​
* 1–5 pills*	103 (67.8%)
* 6–10 pills*	17 (11.2%)
* 11–19 pills*	8 (5.3%)
* ≥ 20 pills*	18 (11.8%)
* Unknown/not reported*	6 (3.9%)
* Self-reported reasons of paracetamol use*	​
* Headache only*	73 (48.0%)
* Headache combined with other reasons*	63 (41.4%)
* Other than headache*	16 (10.5%)
Dipyrone (number of users)	37
* Total number of pills up to study enrollment*	​
* 1–5 pills*	21 (56.8%)
* 6–10 pills*	3 (8.1%)
* >10 pills*	1 (2.7%)
* Intravenous use*	5 (13.5%)
* Unknown/not reported*	7 (18.9%)
* Self-reported reasons of dipyrone use*	​
* Headache only*	18 (48.6%)
* Headache combined with other reasons*	9 (24.3%)
* Other than headache*	7 (18.9%)
* Missing*	3 (8.1%)
Ibuprofen (number of users)	33
* Total number of pills up to study enrollment*	​
* 1–5 pills*	21 (63.6%)
* 6–10 pills*	6 (18.2%)
* >10 pills*	3 (9.1%)
* Unknown/not reported*	3 (9.1%)
* Self-reported reasons of ibuprofen use*	​
* Headache only*	12 (36.4%)
* Headache combined with other reasons*	11 (33.3%)
* Other than headache*	10 (30.3%)

### 3.3 Predictors of analgesic use

Associations between sociodemographic and lifestyle factors and the use of any analgesic or paracetamol were assessed by univariate and multivariate logistic regression. The self-reported health status was associated with the use of any analgesic and paracetamol in both univariate and multivariate models. In the adjusted models, the odds of using any analgesic or paracetamol were significantly higher in women reporting fair/poor health than in those with good/excellent health perception (Tables 4, 5). The gestational age was also positively associated with the use of any analgesic. Multicollinearity among independent variables described in Tables 4, 5 was checked by calculation of variance inflation factors (VIF) using linear regression models. All VIF values were below 1.012, indicating no relevant multicollinearity.

**TABLE 4:** Univariate and multivariate analysis of sociodemographic and lifestyle factors associated with early pregnancy use of any analgesic.

Variables	Use of any analgesic^a^		Crude OR (95% CI)	p-value	Adjusted OR (95% CI)	p-value
	No (sample size)	Yes (sample size)				
Age (years)	26.9 ± 6.2 (106)	27.6 ± 6.1 (169)	1.03 (0.99–1.07)	0.210	1.04 (0.99–1.08)	0.113
Gestational age (weeks)	11.4 ± 3.0 (106)	12.1 ± 2.9 (169)	1.08 (0.99–1.18)	0.062	**1.12 (1.02–1.23)**	**0.016**
Pre-pregnancy BMI (kg/m^2^)	24.5 ± 5.5 (101)	25.6 ± 4.6 (167)	1.05 (0.99–1.10)	0.081	1.05 (0.99–1.11)	0.085
Self-reported health status	38.5% (106)	61.5% (169)	​	​	​	​
* Good/excellent*	42.3% (94)	57.7% (128)	Reference	​	Reference	​
* Fair/Poor*	22.6% (12)	77.4% (41)	**2.51 (1.25–5.03)**	**0.010**	**3.05 (1.44–6.50)**	**0.003**
Race	39.3% (106)	60.7% (164))	​	​	​	​
* White*	42.3% (74)	57.7% (101)	Reference	​	Reference	​
* Other*	33.7% (32)	66.3% (63)	1.44 (0.86–2.43)	0.168	1.43 (0.82–2.50)	0.204
Use of other pharmaceuticals^b^	​	​	​	​	​	​
* No*	43% (61)	57% (81)	Reference	​	Reference	​
* Yes*	33.8% (45)	66.2% (88)	1.47 (0.93–2.40)	0.121	1.27 (0.75–2.13)	0.378

OR, odds ratio; CI, confidence interval. Adjusted models included all variables in the Table. Sample size of adjusted model = 263. Bold values indicate significant associations.

^a^ Data represent mean ± standard deviation for continuous variables and percent count for categorical variables.

^b^ Use of any pharmaceuticals other than analgesics.

**TABLE 5:** Univariate and multivariate analysis of sociodemographic and lifestyle factors associated with early pregnancy use of paracetamol.

Variables	Use of paracetamol^a^		Crude OR (95% CI)	p-value	Adjusted OR (95% CI)	p-value
	No (sample size)	Yes (sample size)				
Gestational age (weeks)	11.5 ± 3.0 (123)	12.0 ± 2.9 (152)	1.06 (0.98–1.15)	0.159	1.08 (0.99–1.18)	0.090
Pre-pregnancy BMI (kg/m^2^)	124.7 ± 5.3 (118)	25.6 ± 4.6 (150)	1.04 (0.99–1.09)	0.175	1.05 (0.99–1.10)	0.097
Self-reported health status						
* Good/excellent*	49.1% (109)	50.9% (113)	Reference	​	Reference	​
* Fair/Poor*	26.4% (14)	73.6% (39)	**2.69 (1.38–5.23)**	**0.004**	**2.84 (1.39–5.79)**	**0.004**
Race						
* White*	48.6% (85)	51.4% (90)	Reference	​	Reference	​
* Other*	40.0% (38)	60.0% (57)	1.42 (0.85–2.35)	0.178	1.31 (0,76–2.28)	0.332
Education						
* Basic*	36.9% (24)	63.1% (41)	Reference	​	Reference	​
* High School*	46.0% (69)	54.0% (81)	0.69 (0.38–1.25)	0.218	0.67 (0.35–1.31)	0.241
* College or higher*	50.0% (30)	50.0% (30)	0.59 (0.29–1.20)	0.142	0.62 (0.280–1.37)	0.239
Use of other pharmaceuticals^b^						
* No*	50.0% (71)	50.0% (71)	Reference	​	Reference	​
* Yes*	39.1% (52)	60.9% (81)	1.56 (0.97–2.52)	0.070	1.45 (0.87–2.42)	0.151

OR, odds ratio; CI, confidence interval. Adjusted models included all variables in the Table. Sample size of adjusted model = 263. Bold values indicate significant associations.

^a^ Data represent mean ± standard deviation for continuous variables and percent count for categorical variables.

^b^ Use of any pharmaceuticals other than analgesics.

**FIGURE 1:**
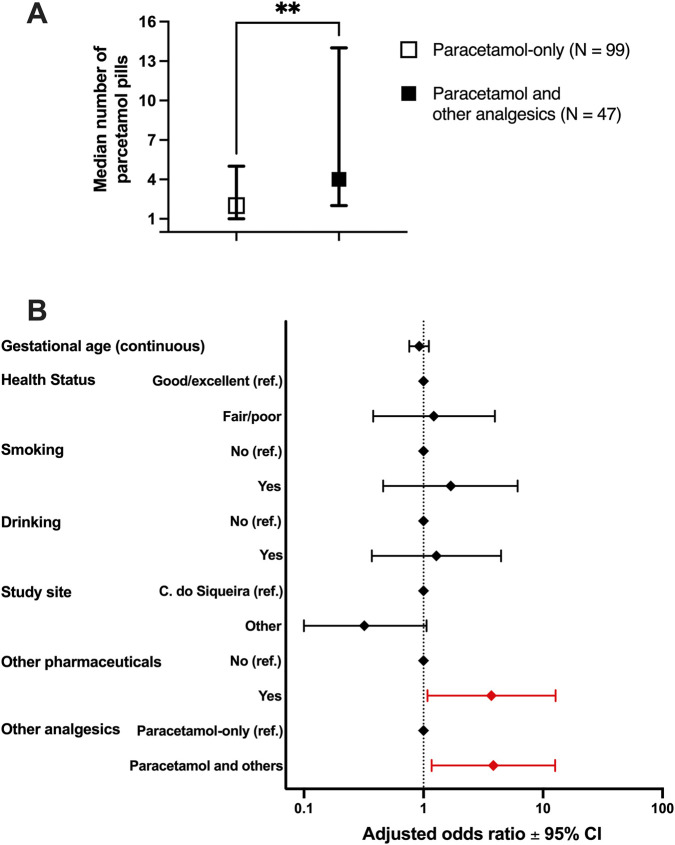
Predictors of total amount of paracetamol consumed in early pregnancy. **(A)** Median number of consumed paracetamol pills among paracetamol-only users and participants who reported consumption of paracetamol and other analgesic types. Data represent median ± interquartile range; **p < 0.01 (Mann-Whitney test). **(B)** Multivariate logistic regression model for the (categorized) outcome amount of paracetamol pills used (1–19 pills/20 or more pills) from pregnancy begin up to study enrollment. Sample size = 145 (1–19 pills = 127; 20 or more pills = 18). Data in red are statistically significant (p < 0.05). All covariates presented were used in the final model. Ref. = reference; CI = confidence intervals.

Among paracetamol users, we also examined the predictors of the amount (number of pills) of paracetamol consumed from the beginning of pregnancy up to study enrollment. The median number of paracetamol pills consumed was significantly higher in women who reported using paracetamol and other analgesics than those who reported using paracetamol-only (Figure 1A). When considering the amount of paracetamol consumed as a categorical variable (1–19 pills/20 or more pills), the odds (95% CI) of being a heavy paracetamol consumer (≥20 pills) was 3.84 (1.17–12.65) times higher in women who reported the use of paracetamol and other analgesics when compared to participants who used paracetamol-only (Figure 1B; Supplementary Table S4). Likewise, the use of pharmaceuticals other than analgesics was significantly associated with the consumption of higher amounts of paracetamol (adjusted OR = 3.70; 95% CI = 1.08–12.74).

## 4 Discussion

The prevalence of analgesic use in our study reveals that 61.5% (N = 169) of the participants reported using at least one analgesic during early pregnancy. Paracetamol was the most used analgesic, reported by 55.3% of women (N = 152), a proportion remarkably consistent with findings from other countries (Werler et al., 2005; Rebordosa et al., 2008; Kristensen et al., 2011; Thorpe et al., 2013; Lind et al., 2017; Bertoldi et al., 2020; Navarro-Lafuente et al., 2021). Many participants also reported the use of other analgesics combination of different types. A strength of our study is that exposure assessments included specific drug names and indications of use. However, medication use was self-reported and may be subject to recall bias, potentially leading to underreporting (Mitchell et al., 1986).

The second most frequently reported analgesic was dipyrone (13.5%), followed by ibuprofen 12% (N = 33), a pattern that likely reflects differences in availability and regulation in Brazil compared with the United States and Western Europe. Although dipyrone has not been consistently associated with major congenital malformations, isolated reports and some epidemiological findings have raised concerns about possible adverse outcomes (Bar-Oz et al., 2005; Da Silva Dal Pizzol et al., 2009; Dathe et al., 2017). Maternal dipyrone use has been associated with the occurrence of oligohydramnios and fetal ductus arteriosus narrowing (Catalán et al., 1995; Sánchez de la Nieta et al., 2003; Weintraub and Mankuta, 2006), as well as increased risk for Wilm’s tumor and leukemia in children (Sharpe and Franco, 1996; Alexander et al., 2001; Couto et al., 2015). Experimental studies from our group suggest potential endocrine-disruptive properties of dipyrone and its main metabolites (Passoni et al., 2018; Passoni et al., 2022). Importantly, the present study was not designed to assess safety outcomes, and no causal inferences can be drawn from these data.

In addition, we assessed associations between the use of analgesics and several sociodemographic and lifestyle factors. As expected, the self-reported health status was a main predictor of any analgesic or paracetamol use in early pregnancy. The odds of using any analgesic or paracetamol were significantly higher in women who reported fair or poor health, compared to participants who reported good or excellent health status. Gestational age at recruitment was also associated with reported analgesic use, likely reflecting longer exposure among women recruited later in early pregnancy. Given the cross-sectional design, these associations should be interpreted cautiously, as temporal relationships cannot be established.

Most women in our study consumed a small number of analgesic pills (1–5 pills), regardless of the analgesic type, and usually to treat headaches. However, 18.5% of participants reported using more than one type of analgesic since the beginning of pregnancy. Among paracetamol users, 11.8% reported consumption of ≥20 pills. Heavy use of paracetamol was significantly associated with the use of additional analgesics and other pharmaceuticals, after adjustment for a set of covariates, including gestational age. This pattern of exposure may reflect a subgroup of pregnant women with more complex health conditions or greater therapeutic needs. However, the fact that pregnant women consuming higher amounts of paracetamol may be more prone to use other analgesics is a cause of concern from a toxicological perspective.

Many of these drugs are suspected of acting as endocrine disruptors that may affect shared signaling pathways and/or negatively impact common target tissues, and together, contribute to the induction of adverse effects during fetal development (Kristensen et al., 2012; Mazaud-Guittot et al., 2013; Ben Maamar et al., 2017; Leverrier-Penna et al., 2018; Rossitto et al., 2019a; Rossitto et al., 2019b; Church et al., 2024; Woodbury et al., 2024). In a study that investigated the potential endocrine disrupting properties of mild analgesics after intrauterine exposure, Kristensen et al. (2011) demonstrated an increased risk of cryptorchidism in sons of women reporting simultaneous use of more than one analgesic when compared to sons of participants who used none or only one analgesic type. In another study, prenatal exposure to paracetamol and other analgesics, but not paracetamol-only, was associated with reduced anoscrotal distance in male infants, a marker of androgen insufficiency during fetal development (Lind et al., 2017). While combined exposure to multiple analgesics has been discussed in the literature in relation to potential endocrine-related effects, our results describe patterns of use only and do not allow conclusions regarding health risks.

Recently, a group of scientists, clinicians, and health professionals published a Consensus Statement on paracetamol use during pregnancy (Bauer et al., 2021). In this Statement, the group raised concerns about the high prevalence of gestational use and potential associations with neurodevelopmental, urogenital, and reproductive disorders. In contrast, recent original studies and systematic reviews have not identified consistent associations between prenatal exposure to paracetamol and neurodevelopmental outcomes in children (Ahlqvist et al., 2024; Sheikh et al., 2025; D'Antonio et al., 2026). Notably, a comprehensive cohort study with sibling control analysis involving over 2.4 million children born in 1995–2019 in Sweden found no associations of *in utero* paracetamol exposure with children’s risk of autism, ADHD, or intellectual disability (Ahlqvist et al., 2024). Although data on these associations are still inconclusive, precautionary actions have been recommended to minimize prenatal exposures to paracetamol, which should be used at the lowest effective dose, for the shortest duration needed, and under medical guidance (Bauer et al., 2021). Our results support the high prevalence of paracetamol use worldwide, including in Brazil, but longitudinal studies with dose-standardized exposure assessment are required to better elucidate possible implications.

This study has several limitations. Analgesic use was self-reported, and exposure was determined by pill counting, which is not dose-standardized and may lead to exposure misclassification. The cross-sectional nature of the study precludes causal inferences. The study population was restricted to women in the public healthcare system, which may limit generalizability. The selection of variables using a p-value threshold in bivariate analysis was employed to avoid overfitting, considering the relatively small sample size; however, this represents an additional limitation. The categorization of continuous variables, which is helpful for interpretation in a small sample, may have led to some loss of information. Results of subgroup analyses, especially among heavier paracetamol users, should be interpreted with caution.

Despite the limitations, our results offer relevant information on the patters of analgesic use during early pregnancy in a Brazilian population. Analgesics, particularly paracetamol, are commonly used, and coexposures are not unusual. These findings point to the need for further research on the potential impact of prenatal exposure to individual analgesics and their combinations on a diverse range of health outcomes, including neurodevelopmental and reproductive effects, as well as for counseling of pregnant women.

## Data Availability

The raw data supporting the conclusions of this article will be made available by the authors, without undue reservation.
