# Supplementary material for: Prevalence and predictors of analgesic use during early pregnancy in a Brazilian population
Source: Front Pharmacol. 2026 Mar 5;17:1730483. doi: 10.3389/fphar.2026.1730483 (PMC12999959; doi:10.3389/fphar.2026.1730483)
Supplement: Supplementary file 1 [file Table1.docx]

**Table S1.** Recruiting study sites – health care units^a^

| **Variables** | **N (%)** |
| --- | --- |
| Participants | 275 |
| Health Care Units |  |
| *Campina do Siqueira* | 79 (28.7%) |
| *Mae Curitibana* | 61 (22.2 %) |
| *Cajuru* | 46 (16.7%) |
| *Other* | 89 (32.4 %) |

^a^ Participating women were recruited in 9 health care units in the city of Curitiba.

**Table S2.** Smoking and drinking habits in study participants

| **Variables** | **N (%)** |
| --- | --- |
| Participants | 275 |
| Smoking | 53 (19.3%) |
| *Less than 1 cigarette/day* | 17 (6.2%) |
| *1-5 cigarettes/day* | 13 (4.7%) |
| *More than 5 cigarettes/day* | 19 (6.9%) |
| *Unknown amount/not reported* | 4 (1.5%) |
| Drinking | 92 (33.5%) |
| *1-5 drinks^a^* | 57 (20.7%) |
| *6-10 drinks^a^* | 15 (5.5%) |
| *More than 10 drinks^a^* | 12 (4.4%) |
| *Unknown amount/not reported* | 8 (2.9%) |

^a^ Total number of standard drinks up to study enrolment. A standard drink is defined as any drink containing 13 g of pure alcohol.

**Table S3.** Early pregnancy use of pharmaceuticals^a^

| **Variable** | **N (%)** |
| --- | --- |
| Participants | 275 |
| *No* | 142 (51.6%) |
| *Yes* | 133 (48.4%) |
| *Antiemetics* | 38 (13.8%) |
| *Antibiotics* | 31 (11.3%) |
| *Thyroid hormones* | 15 (5.5%) |
| *Progestins* | 13 (4.7%) |
| *Antidepressants* | 10 (3.6%) |
| *Spasmolytics* | 10 (3.6%) |
| *Other* | 46 (16.7%) |

^a^ Self-reported use of any pharmaceutical (except analgesics) up to enrolment. Some participants reported the use of more than one pharmaceutical class.

**Table S4.** Univariate and multivariate analysis of predictors of paracetamol amount used in early pregnancy

| **Variables** | **Number of paracetamol pills^a^** | | **Crude OR (95% CI)** | **p-value** | **Adjusted OR (95% CI)** | **p-value** |
| --- | --- | --- | --- | --- | --- | --- |
|  | **1-19 (sample size)** | **≥ 20 pills (sample size)** |  |  |  |  |
| Gestational age (weeks) | 12.0 ± 2.8 (128) | 11.2 ± 3.6 (18) | 0.91 (0.77 – 1.07) | 0.246 | 0.92 (0.76 – 1.11) | 0.398 |
| Self-reported health status | 87.7% (128) | 12.3% (18) |  |  |  |  |
| *Good/excellent* | 89.7% (96) | 10.3% (11) | Reference |  | Reference |  |
| *Fair/Poor* | 82.1% (32) | 17.9% (7) | 1.09 (0.68 – 5.34) | 0.218 | 1.22 (0.38 – 3.96) | 0.740 |
| Smoking | 87.6% (127) | 12.4% (18) |  |  |  |  |
| *No* | 89.7% (105) | 10.3% (12) | Reference |  | Reference |  |
| *Yes* | 78.6% (22) | 21.4% (6) | 2.39 (0.81 – 7.04) | 0.115 | 1.69 (0.46 – 6.13) | 0.428 |
| Drinking | 87.6% (127) | 12.4% (18) |  |  |  |  |
| *No* | 90.6% (87) | 9.4% (9) | Reference |  | Reference |  |
| *Yes* | 81.6% (40) | 18.4% (9) | 2.18 (0.80 – 5.89) | 0.127 | 1.28 (0.37 – 4.45) | 0.699 |
| Study site | 87.7% (128) | 12.3% (18) |  |  |  |  |
| *Campina do Siqueira* | 80.0% (36) | 20.0% (9) | Reference |  | Reference |  |
| *Other* | 91.1% (92) | 8.9% (9) | 0.39 (0.14 – 1.07) | 0.066 | 0.32 (0.10 – 1.06) | 0.063 |
| Other pharmaceuticals^b^ | 87.7% (128) | 12.3% (18) |  |  |  |  |
| *No* | 94.1% (64) | 5.9% (4) | Reference |  | Reference |  |
| *Yes* | 82.1% (64) | 17.9% (14) | **3.5 (1.09 – 11.21)** | **0.035** | **3.70 (1.08 – 12.74)** | **0.038** |
| Other analgesics | 87.7% (128) | 12.3% (18) |  |  |  |  |
| *Paracetamol-only* | 91.9% (91) | 8.1% (8) | Reference |  | Reference |  |
| *Paracetamol and others* | 78.7% (37) | 21.3% (10) | **3.07 (1.13 – 8.40)** | **0.029** | **3.84 (1.17 – 12.65)** | **0.027** |

OR = odds ratio; CI = confidence interval. Adjusted models included all variables in the Table. Sample size of adjusted model = 145. Significant associations are marked in bold. ^a^ Data represent mean ± standard deviation for continuous variables and percent count for categorical variables. ^b^Use of any pharmaceuticals other than analgesics.
